# HEALTH AND WELL-BEING OUTCOMES OF THE OASIS INTERGENERATIONAL TUTORING PROGRAM

**DOI:** 10.1093/geroni/igad104.1444

**Published:** 2023-12-21

**Authors:** Peter Sun, Nancy Morrow-Howell

**Affiliations:** Washington University in St. Louis, St. Louis, Missouri, United States; Washington University in St. Louis, St. Louis, Missouri, United States

## Abstract

The Oasis Intergenerational Tutoring Program is a national program that connects older adult tutors with elementary children in Grades K-3. We are conducting an ongoing project to assess multiple health and well-being outcomes associated with this tutoring program, especially among four sub-groups: first-time tutors, male tutors, tutors who are caregivers, and tutors who report being lonely. In year one of our project, we have collected data from 329 older adults who tutored children in the 2021-2022 school year. We found that a larger proportion of tutors who felt lonely reported improvements to their health, compared to tutors who did not feel lonely. We also found that first-time tutors experienced larger increases in at least two areas of health (physical, emotional, and cognitive), compared to repeat tutors. When we asked tutors about their experience tutoring during the pandemic, major themes included a sense of purpose in life, increased social engagement, and the negotiation of risks and benefits (e.g., remote vs. in-person tutoring). An upcoming two-year follow-up study will enable us to compare our findings with the Health and Retirement Study, a nationally representative sample of older adults. Findings from this project will inform programs across the nation and policies that seek to harness the power of intergenerational bonds.

